# Smallholders’ knowledge about healing goat gastrointestinal parasite infections with wild plants in southern DR Congo

**DOI:** 10.3389/fphar.2023.1124267

**Published:** 2023-03-01

**Authors:** Gaël Nzuzi Mavungu, Cedrick Shakalenga Mutombo, Désiré Mujike Numbi, Salvatora Nkulu Nsenga, Welcome Nonga Muyumba, Celestin Shongo Pongombo, Salvius Amuri Bakari, Amandine Nachtergael, Sandrina Vandenput, Victor Embeya Okombe, Pierre Duez

**Affiliations:** ^1^ Faculty of Veterinary Medicine, Unit of Pharmacology and Therapeutic, University of Lubumbashi (UNILU), Lubumbashi, Congo; ^2^ Unit of Therapeutic Chemistry and Pharmacognosy, Université de Mons (UMONS), Mons, Belgium; ^3^ Faculty of Veterinary Medicine, Université de Liège (ULiège), Fundamental and Applied Research for Animals & Health (FARAH), Liege, Belgium; ^4^ Laboratory of Pharmacognosy, Faculty of Pharmaceutical Sciences, University of Lubumbashi (UNILU), Lubumbashi, Congo; ^5^ Faculty of Agronomic Sciences, University of Lubumbashi (UNILU), Lubumbashi, Congo; ^6^ Department of Chemistry-physics Lubumbashi, High college training teachers of Lubumbashi, Lubumbashi, Congo

**Keywords:** goat keepers, herbal remedies, palmately compound leaves, gastrointestinal parasites, ethnoveterinary knowledge, Lubumbashi

## Abstract

Gastrointestinal parasite (GIP) infections control has an important role to play in increasing livestock production from a limited natural resource base and to improve animal health and welfare. This study aimed to collect indigenous knowledge and identify wild plants locally used by goat smallholders of three territories of Haut-Katanga province for treating signs of gastrointestinal parasitism. Ethnoveterinary surveys were conducted by semi-structured interviews and a bibliographic screening of the biological activities relating to cited plants was carried out. Our interviews showed that ethnosemantic diagnoses of GIP diseases are based on signs. Eighty-seven informants reported that 27 plant species from 15 families, dominated by Fabaceae (29.6%) and Lamiaceae (18.5%) were commonly used in their goats treatment. Among these plants, five species with palmately compound leaves were considerably more used. From those, we noted a substitution of *Vitex congolensis* De Wild. and T. Durand (Lamiaceae) by *Oldfieldia dactylophylla* (Welw. Ex Oliv.) J. Leonard (Picrodendraceae) and of *Vitex mombassae* Vatke by *Vitex madiensis* Oliv. Subsp. *Milanjiensis* (Britten) F. White. Roots (46.9%), leaves (28.0%) and seeds (12.5%) were the most frequently used plant organs, and maceration is applied for most of the medicinal preparations (62.2%). Recipes were administered by oral route, for GIP 1) prevention (33.3%), by macerating the ground plant material in drinking water for 2 weeks at the start of each season (dry and rainy); and 2) treatment (66.7%). According to the literature, some of these plants have few or no studies investigating their anthelmintic activity. The cited plants are worth investigating further as they could constitute an effective alternative strategy in maintaining animal productivity. Studies on the biological activity of these plants can also provide indications of promising leads for extracts that could be developed into commercial standardized medications.

## 1 Introduction

Livestock play a significant role in livelihoods and the economies of developing countries (Herrero et al., 2013; Hatab et al., 2019). In DR Congo, small livestock production systems are primordial for meat production and to generate income (Wasso et al., 2018; Klapwijk et al., 2020). In Haut-Katanga province, in rural or peri-urban contexts, animal raising is a familial activity, that involves a small number of animals per family. The most common species are poultry (chicken and ducks), goats and sometimes one or two pigs, usually free roaming (Ngona, 2008; Kalenga et al., 2015). Families do not spend much time or money on their livestock: this certainly explains why goats are so widespread and why it plays an important socio-economic role for meat production and to generate income. These livestock keeping are carried out without adequate veterinary advice and with little or no knowledge of goat diseases. In addition, livestock keepers have little access to medicines (Gemeda et al., 2020) and many treat symptoms of disease with herbal remedies. Gastrointestinal parasite (GIP) infection remains one of the main health (Mpofu et al., 2020) and production constraints of goats as they cause severe injuries to infected animals and significant losses in farming production worldwide (Zvinorova et al., 2016).

A survey has already been conducted in rural area in the regions of Kamina and Kaniama (600 km northwest of Lubumbashi, DR Congo) to identify traditional herbal remedies used in the control of gastrointestinal disorders in ruminants (Okombe et al., 2014a). This study revealed that livestock keepers treat when they observe direct signs (presence of worms in feces) or indirect signs in their goats (rough hair coat, loss of weight or appetite, distended abdomen or diarrhea). From this study, the root bark powder of *Oldfieldia dactylophylla* (Welw. Ex Oliv.) J. Leonard (misidentified as *Vitex thomasii* De Wild) was the most widely used by farmers. However, little is known about the ethnoveterinary (EV) use of medicinal plants to control GIP infections in goats in the province of Haut-Katanga, which is characterized by different climatic conditions and floristic diversity from the aforementioned regions (Meerts, 2016).

Through a survey of goat farmers who treat their animals with herbal remedies that they make themselves, the objective of the study was to identify the plants used to make these remedies and to verify whether the literature documents their therapeutic usage and/or effectiveness. This information is needed to collect data on EV practices and to consider efficacy studies of plants that are used in field. If, in the long run, studies prove the effectiveness of these therapeutic uses, it is an opportunity for Congolese herders who do not have easy access to drug treatments.

## 2 Material and methods

### 2.1 Study design and description of the study area

**FIGURE 1:**
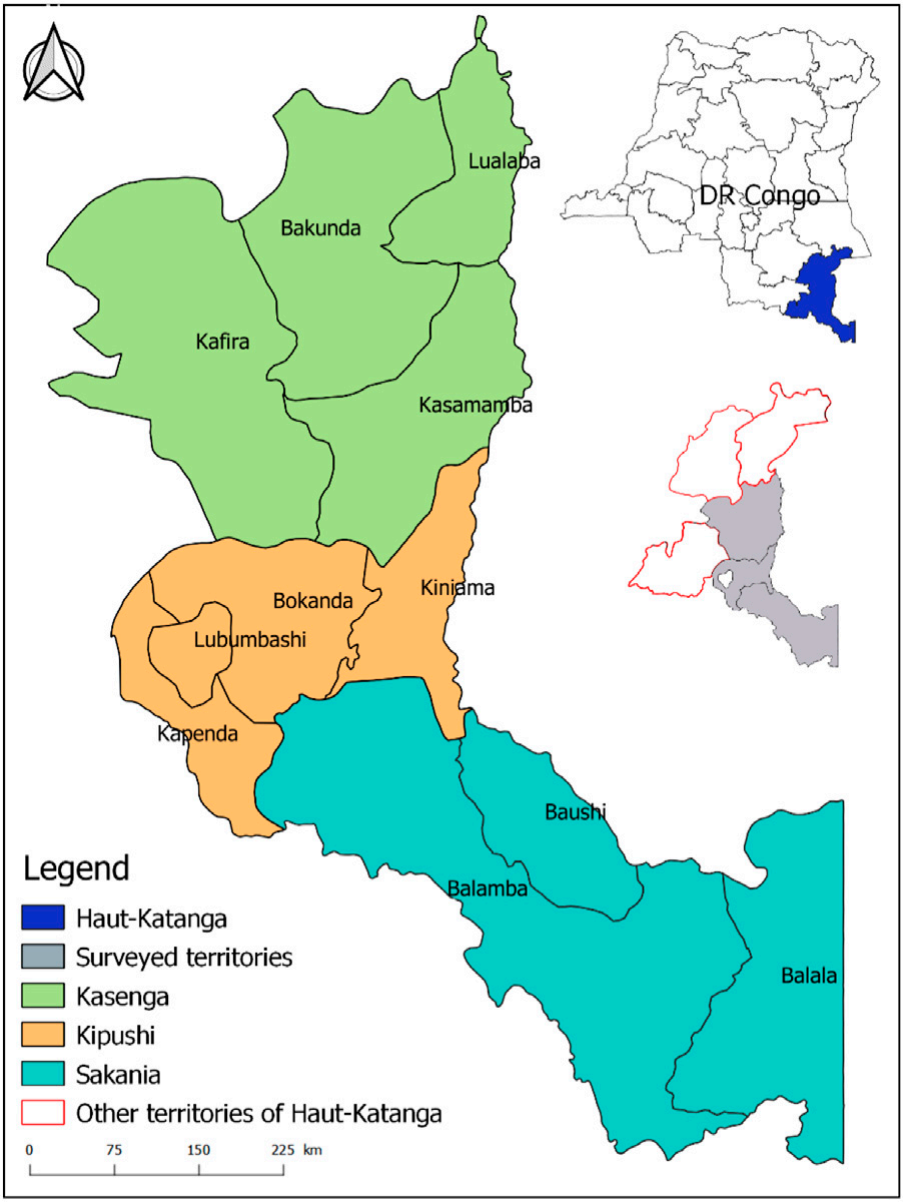
Map of the study area and its location in DR Congo.

The cross-sectional survey was conducted in the Haut-Katanga province, south-eastern of the DR Congo (Figure 1). This province lies between 27°30′ and 29°30′ east longitude and between 7°15′ and 13°30′ south latitude with a total area of 134,431 km^2^. There is a tropical climate in this region with a rainy (November to March) and a dry season (April to October). The annual average temperatures vary between 14.2°C and 24.6°C, while the average maxima is between 25°C and 32°C (Malaisse, 1997).

The province of Haut-Katanga is mainly occupied by shrubby savannah in the northwest, a clear forest and a wooded savannah in the northeast and southeast, then a covering of wooded and tree savannah in the southwest (De Weerdt et al., 2018). Lubumbashi is the capital and principal city of the province with a current population estimated at 2,811,959 in habitants (https://worldpopulationreview.com/world-cities/lubumbashi-population). Haut-Katanga is one of the world’s largest producers of heavy metals, particularly copper, cobalt and manganese, so that its economic growth is essentially based on the exploitation of its mines (De Weerdt et al., 2018). With six territories in total in this province, this study was conducted in three of its territories, i.e. Kasenga (10°21′47″S; 028°36′54.5″E; average altitude, 955 m), Sakania (12°44′59.6″S; 028°33′34″E; average altitude, 1,305 m) and Kipushi (11°46′1.24″S; 27°13′58.8″E; average altitude, 1,329 m). These territories, selected for their easy access by road from Lubumbashi, include many localities which are generally classified as rural and where conventional drugs are either unavailable or unaffordable to livestock keepers.

### 2.2 Recruitment and study population characteristics

For each survey area, the local administrator of the Rural Development Department was asked to report on goat keepers who use plant remedies for their animals. These goat keepers were contacted by the surveyor. The eligibility criteria for participation in the survey were adults that had at least one goat and treat animal(s) with remedies made by themselves or a family member.

The sociodemographic situation of interviewed goat keepers is summarized in Table 1 **.** Most of them were men, ranging in age from 20 to 72 years, with a median value of 46 years. Regarding educational status, mostly non-educated (55.2%) and elementary school-level (23.0%) informants reported knowledge on veterinary traditional medicine. Most of participants (46.0%) acquired traditional knowledge from their families, 31.0% from friends and other livestock keepers, 12.6% from personal experience in animal husbandry and 10.4% from informal training in human traditional medicine. In this study, 69.0% of goat keepers stated applying traditional treatments on their animals for ≥10 years, some claiming up to 60 years’ experience.

**TABLE 1:** Sociodemographic characteristics of surveyed goat keepers (n = 87).

Characteristics	Category level	Frequency	Percentage (%)
Sex	Female	16	18.4
	Male	71	81.6
Age	20–30	10	11.5
	31–45	15	17.2
	46–72	62	71.3
Educational level	Illiterate	42	55.2
	Elementary school	23	23.0
	Secondary education	19	18.4
	University	3	3.4
Source of ethnoveterinary healing knowledge	Parents or family members	40	46.0
	Close friends and other livestock keepers	27	31.0
	Personal experience	11	12.6
	Formation	9	10.4
Level of ethnoveterinary practice experience (year)	<10	27	31.0
	10–20	9	10.4
	21–35	21	24.1
	36–60	30	34.5

### 2.3 Data collection methods and survey administration

In accordance with the eligibility criteria, of the 139 goat keepers invited to participate in the survey and the collect of plants, 87 agreed to collaborate and were interviewed. Semi-structured questionnaires were used to obtain information in an in-depth interview with study participants. They were conducted over a period of 6 months, from September 2017 to March 2018. The questionnaire was not pretesting on this population, but it is similar to those used by Okombe et al. (2014a) and Tufa et al. (2019). The questionnaire was prepared in French and administered in local language (Swahili) with the help of local translators. An average interview lasted 30 and 45 min. Collected data were informations on the administered therapies i.e., recognized symptoms, local names of plants, plant parts used, recipes, methods of preparation, dosages, routes of administration and the duration of treatments.

### 2.4 Ethical statement

The study protocol was realized in accordance with the *ethical principles for medical research involving human subjects* adopted by the Declaration of Helsinki, article 24 (WMA, 2015). Each respondent had received explanation about the objectives of the study and was free to participate to the survey. During data collection, an effort was made verbally to encourage livestock keepers by explaining that their cooperation benefits the country and that disclosure of their knowledge of medicinal plants would not disrupt their practices.

The collection of the plant material was done under the guidance of one of the community leaders, ensuring that the biodiversity rights of the indigenous people were protected as data were acquired under the *Nagoya Protocol* (National authorization N° 745/CAB/MIN/EDD/AAN/TNT/SAA/2018, covering the PhytoKat project, 2017-2022).

### 2.5 Collection and identification of plant material

Following the interviews, the cited medicinal plants were collected in the field in the presence of the goat keeper who uses them in his recipes. The local name, collection date, collection site, and series number were noted. The medicinal plants were harvested in sufficient quantities for *i)* preliminary identification by botanist researchers from the Laboratoire d’Ecologie, Restauration écologique et Paysage at the University of Lubumbashi (D. Mujike and M. Nkulu); and *ii)* confirmation at the herbarium of INERA-Kipopo, Lubumbashi. The collected plant samples were identified with the help of the African Plant Database (CJB, 2012) and finally scientific names were verified and corrected through The Herbarium Catalogue, Royal Botanic Gardens, Kew (http://www.plantsoftheworldonline.org/). Each sample was appropriately dried and pressed for conservation, following international standards (Stuessy and Sohmer, 1996).

### 2.6 Literature-based validation of collected data

For the most used medicinal plants species, a systematic literature search of ethnobotanical information was conducted in Medline^®^ (Pubmed^®^ interface), CAB Abstracts^®^, https://explore.lib.uliege.be/discovery/dbfulldisplay?docid=alma9919425387202321&context=L&vid=32ULG_INST:ULIEGE&lang=fr&adaptor=Local%20Search%20Engine&tab=jsearch_slot&query=contains%2Cdbcategory%2C&sortby=title&offset=0&databases=category,MEDE Allied and Complementary Medicine : AMED^®^ and Scopus^®^. All original articles published on their anthelmintic traditional uses, pharmacological activities and chemical constituents were considered for inclusion.

### 2.7 Data management

The network visualizing plant uses in the three study territories was generated using Cytoscape 3.8.0 software (https://cytoscape.org/download.html), with an organic layout, according to Mukazayire et al. (2011).

## 3 Results

### 3.1 Information on administered therapies

The results of the survey on veterinary herbal therapy in goats in the Haut-Katanga province are summarized in Table 2.

**TABLE 2:** Plants used against gastrointestinal parasite infections in goats from 3 territories of the Haut-Katanga province: Overview of herbal medicines collected and identified.

Scientific name	VSN^a^	Family name	Vernacular name	Plant habit ^b^	Source ^c^	Plant parts used, preparation and mode of administration	Diagnosis of worm infection	Type of treatment	Citation number (n = 87)
*Oldfieldia dactylophylla* (Welw. Ex Oliv.) J.Leonard	MM 028-ECO	Picrodendraceae	Kikoto muchi	T	W	Dried root barks grinded to powder and macerated with water is given orally to the sick animal, 1 glass 1 time per day for 3 days. Roots are used in decoction and drunk, 2 glasses for 2-3 days	Presence of worms and blood in feces	Curative care	40
*Vitex madiensis* Oliv. Subsp. *Milanjiensis* (Britten) F.White	DM 003-ECO	Lamiaceae	Mufutu	T	W	The root barks are used in maceration or decoction and drunk, 1 glass per day for 2-3 days	Vomiting, anorexia	Curative care	39
*Vitex fischeri* Gürke	DM 002-ECO	Lamiaceae	Mufutu	T	W	The root barks are used in maceration or decoction and drunk, 1 glass per day for 2-3 days	Bloated abdomen, anorexia	Curative care	38
*Vitex doniana* Sweet	DM 001-ECO	Lamiaceae	Mufutu	T	W	The root barks are used in maceration or decoction and drunk, 2 glasses per day for 2-3 days	Presence of worms and blood in feces	Curative care	32
*Vitex mombassae* Vatke	DM 004-ECO	Lamiaceae	Mufutu	T	W	The root barks are used in maceration or decoction and drunk, 2 glasses per day for 2-3 days	Presence of worms and blood in feces	Curative care	30
*Moringa oleifera* Lam	MaMu 023-FSA	Moringaceae	Muringa	T	C	The seeds are crushed and mixed with food and then fed to the animal, for 7 days. Root barks are crushed and diluted in water for 7 days. Leaves are directly consumed by animals	Pitted hairs	Preventive care	24
*Cassia abbreviata* Oliv	MaMu 009-FSA	Fabaceae	Kafungunansha	T	W	The root barks of the plant are crushed and macerated in water and drunk, 1/2 glass 1 time per day for 2 days	Weight loss and anorexia	Curative care	23
*Carica papaya* L		Caricaceae	kipayipayi	T	C	Dried seeds grinded to powder and macerated with water is given in oral to the sick animal, 1 glass, 1 time per day for 3 days	Weight loss and anorexia	Curative care	21
*Aloe buettneri* A. Berger	MaMu 002-FSA	Asphodelaceae	Iposo	H	W	Leaves are used in decoction or maceration and drunk by the sick animals, 1/3 glass, 1 time per day for 2 days	Weight loss and anorexia	Curative care	19
*Tephrosia vogelii*Hook.f	MaMu 032-FSA	Fabaceae	Buba	S	W	Crushed fresh leaves used in maceration or decoction with water to drink, 1 glass per day for 2-3 days	Bloated abdomen, anorexia	Curative care	19
*Tetradenia riparia* (Hochst.) Codd	MaMu 034-FSA	Lamiaceae	Mutuzo	S	C	Crushed fresh leaves used in decoction with water to be drunk, 1/2 glass one a day for 2 days	Presence of worms and blood in feces	Curative care	19
*Bobgunnia madagascariensis* (Desv.) J.H.Kirkbr. and Wiersema	MaMu 007-FSA	Fabaceae	Ndale	T	W	Crushed seeds or root are dilute in water then wait for 3 days before administration, 1 glass for 2 days	Vomiting, anorexia	Curative care	18
*Allium sativum* L		Amaryllidaceae	Matungulu ayi	H	C	Bulbs are crushed, macerated or infused in water and drunk by the sick animal, 1/2 glass, 2 times a day for 2 days	Presence of worms and blood in faeces	Curative care	17
*Hymenocardia acida* Tul	MaMu 019-FSA	Phyllanthaceae	Kapempe	T	W	Crushed fresh leaves homogenized in water to drink, 1 glass per day for 3 days	Presence of worms and blood in feces	Curative care	17
*Terminalia mollis* M.A. Lawson	MaMu 033-FSA	Combretaceae	Kibobo	T	W	Crushed fresh leaves or root barks used in maceration or decoction with water to drink, 1 glass per day for 3 days	Presence of worms and blood in feces	Curative care	17
*Parinari curatellifolia* Planch. Ex Benth	MaMu 029-FSA	Chrysobalanaceae	Mupundu	T	W	Crushed fresh root homogenized in water to drink, 1/2 glass per day for 3 days	Presence of worms and blood in feces	Curative care	16
*Allium cepa* L		Amaryllidaceae	Matungulu	H	C	Bulbs are crushed, macerated in water and drunk by the sick animal, 1 glass 2 times a day for 2 days	Presence of worms and blood in faeces	Curative care	15
*Cucurbita moschata* Duchesne	MaMu 015-FSA	Cucurbitaceae	Kiboke	H	C	Dried and crushed seeds are prepared in decoction with water. The liquid is given to be drunk to the animal, 2 glasses a day for 2-3 days	Vomiting, anorexia	Curative care	14
*Kigelia africana* (Lam.) Benth	MaMu 022-FSA	Bignoniaceae	Kamfungwila	T	W	The inner part of the fruit or the root barks are homogenized in water to drink. The maceration is left permanently in a drinker for 10 days	Diarrhea	Preventive care	12
*Annona senegalensis* Pers	MaMu 005-FSA	Annonaceae	Mulolo	T	W	Fresh crushed leaves are homogenized in water to drink. The maceration is left permanently in the drinker for 7 days	Pitted hairs, diarrhea	Preventive care	10
Isoberlinia angolensis (Welw. Ex Benth.) Hoyle & Brenan	MaMu 020-FSA	Fabaceae	Mutobo	T	W	Crushed fresh root homogenized in water to drink, 1 glass 2 times a day for 7 days	Vomiting	Preventive care	10
Pterocarpus tinctorius Welw	MaMu 031-FSA	Fabaceae	Mukula	T	W	Crushed fresh root homogenized in water to drink, 1 glass per day for 7 days	Pitted hairs, weight loss	Preventive care	10
Dialium angolense Welw. Ex Oliv	MaMu 016-FSA	Fabaceae	Mupepetwa	T	W	Crushed fresh leaves homogenized in water to drink. The maceration is left permanently in a drinker for 5 days	Vomiting	Preventive care	9
Dysphania ambrosioides (L.) Mosyakin & Clemants	MaMu 012-FSA	Amaranthaceae	Lufwa nyoki	H	W	The leaves are used in decoction and drunk 1 glass per day for 5 days	Weight loss during the rainy season	Preventive care	8
Mucuna pruriens L.) DC.	MaMu 024-FSA	Fabaceae	Msepe	H	W	Crushed fresh root homogenized in water to drink. The maceration is left permanently in a drinker for 7 days	Diarrhea	Preventive care	8
*Balanites aegyptiaca* L.) Delile	MaMu 006-FSA	Zygophyllaceae	Mubamb wa ngoma	T	W	Stem bark is used in decoction. The cooled liquid is drunk by the animal, 1 glass, for 2-3 days	Presence of worms and blood in feces, anorexia	Curative care	5
*Julbernardia paniculata* (Benth.) Troupin	MaMu 021-FSA	Fabaceae	Mutondo	T	W	Crushed fresh root homogenized in water to drink. The maceration is left permanently in the drinker for 7 days	Diarrhea	Preventive care	5

^a^ VSN, voucher specimen number.

^b^ T = tree, H = herb, S = shrub.

^c^ C = cultivated; W = wild.

Decoction (plant material is boiled with water); maceration (plant material is soaked for a specified time in water, room temperature); infusion (the plant material is soaked in boiling water in the state while the water is cooling); in any case, the liquid is usually decanted and sometimes filtered through a sieve.

#### 3.1.1 Taxonomy, plant parts used and identification of gastrointestinal parasitism

**FIGURE 2:**
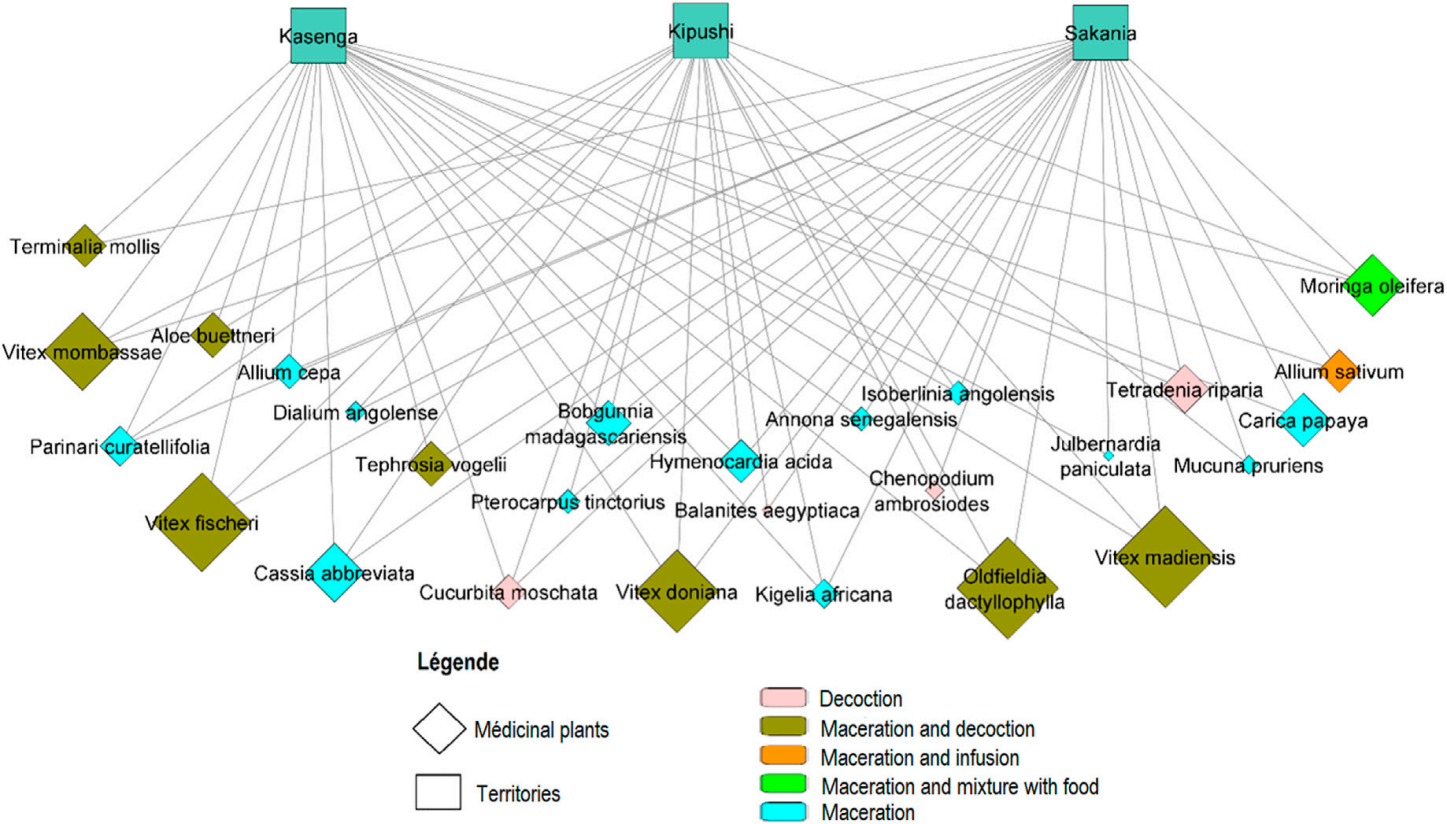
On this graph, territories are represented by squares, while diamonds represent plants used. Methods used for the preparation of traditional herbal remedies are represented by the different colors of the diamonds. The sizes of the diamonds are proportional to the frequencies of use; the larger ones represent the most used plants.

A total of 27 different plant species, belonging to 15 botanical families, were recording during the 87 interviews (Table 2). Fabaceae family species were the most frequently used (29.6%), followed by Lamiaceae (18.5%). An average of 5.5 ± 2.5 plants were cited per respondent for a citation number ranging from 1 to 15 (Figure 2).

Our results showed that five palmately compound leaf species were considerably more used; these are *Oldfieldia dactylophylla* (Picrodendraceae) (8.1%) and *Vitex fischeri* Gürke (7.7%), *Vitex madiensis* Oliv. Subsp. *Milanjiensis* (Britten) F. White with 7.9%, *Vitex doniana* Sweet (6.5%) and *Vitex mombassae* Vatke (6.1%) (Lamiaceae).

The taxonomic verification of the species vernacular names revealed that, among the most widely used plants, some were substituted and/or misidentified by users. This was the case for *O. dactylophylla* which is confused with *Vitex congolensis* and for *V. madiensis*, confused with *V. mombassae*. Within some of these species, different morphotypes have been identified, relating to the number of leaflets. This was the case with *V. madiensis* and *V. mombassae* for which leaves can be composed by either 3 leaflets or 3 to 5 leaflets.

Among the recorded species, trees were the largest category (70.4%), followed by herbs (22.2%) and shrubs (7.4%).

Roots were the most frequently used plant organs (46.9%), followed by leaves (28.0%). Other organs reported to be used were seeds (12.5%), bulbs (6.3%), stems (3.1%) and fruits (3.1%).

The informants reported that they decide to treat animals showed the following signs: presence of worms and/or blood in feces, weight loss, anorexia, pitted hairs, diarrhea, vomiting, bloated abdomen and cough.

#### 3.1.2 Preparation and administration of remedies

For treatment of worm infections in goat, the medicinal plants were prepared and administered orally, mostly as a maceration (62.2%), a decoction (32.4%) or an infusion (2.7%), using water as solvent. In other cases (2.7%), the remedies were prepared by pounding the plant part and mixing with food. Treatment could take longer for preventive care (33.3%) than for healing (66.7%). All cited remedies were composed of a single herb. No toxicity or adverse effects have been reported by interviewed respondents.

### 3.2 Bibliographic data on the anthelmintic activities of most cited plants

The collected bibliographic data presented in Table 3 revealed that the five plants mostly used by livestock keepers for the control of GIP in goats (*O. dactylophylla*, *V. fischeri*, *V. madiensis*, *V. doniana* and *V. mombassae*) have not yet been thoroughly investigated. For *O. dactylophylla*, only three publications concerned its traditional use as an anthelmintic (of which two were published under the wrong name of *Vitex thomasii*, which we could verify by examination of the voucher specimens cited in these reports) and there are so far no biological studies on the possible activities of *V. fischeri* and *V. mombassae* against gastrointestinal parasitosis.

**TABLE 3:** Litterature screening for anthelmintic properties of the 5 plants most cited by goat keepers for the control of gastrointestinal parasite diseases.

Scientific name	Previously reported traditional uses	Reported pharmacology activities	Chemical constituents
*Oldfieldia dactylophylla* (Welw. Ex Oliv.) J.Leonard	The root bark is crushed and macerated for use as an enema against gastrointestinal worms Okombe et al. (2014b) ^a^, Novotna et al. (2010)	*In vivo* antiparasitic efficacy of the root bark powder on the digestive strongyles of grazing goats Okombe (2011)	Tannins, flavonoids, iridoids, triterpenoids Okombe (2011)
*Vitex doniana* Sweet	Use of leaves in the control of worm infections in poultry Alawa et al. (2002), N’Danikou et al. (2015), Dansou et al. (2021)	*In vitro* anthelmintic activity on *Caenorhabditis elegans* Waterman et al. (2010); *in vitro* egg hatch inhibition in *C. elegans* Gagman et al. (2019)	Anthraquinones, flavonoids, saponins, sterols, tannins and phenols Suleiman and Yusuf (2008), Novotna et al. (2010)
*Vitex fischeri* Gürke	No report	No report	No report
*Vitex madiensis* Oliv. Subsp. *Milanjiensis* (Britten) F.White	Anthelmintic properties Lengbiye et al. (2018)	*In vitro* anthelmintic activity against earthworms Lengbiye et al. (2020)	Flavonoids and polyphenols, terpenoids, tannins and organic acids Lengbiye et al. (2018)
*Vitex mombassae* Vatke	No report	No report	No report

^a^ The study investigated *Oldfieldia dactylophylla* under the wrong name of *Vitex thomasii* De Wild, which we could verify by examination of the cited voucher specimens.

## 4 Discussion

The present study identified 27 medicinal plants that are used by goat keepers in the Haut-Katanga province to treat signs of GIP infections in goats. From the identified medicinal plant (Table 2), Fabaceae was the most common plant family reported, followed by Lamiaceae. In agreement with our study, these botanical families have previously been reported in this region as the dominant plant families used in traditional medications (Bakari et al., 2017; Chiribagula et al., 2017). This might be correlated with the abundance of species belonging to these families in the study area.

Of the cited species, five of them, belonging to the family of Picrodendraceae (*O. dactylophylla*) and Lamiaceae (*V. doniana, V. fischeri, V. madiensis* and *V. mombassae*), were considerably more used. The frequency of their uses could in fact be related to its effectiveness as a remedy, to its availability in the study area (Ahmad et al., 2014; Amjad et al., 2017) or to taxonomic confusions. These plants are all morphologically characterized by their palmately compound leaves. The substitution observed in identifying these species may result in diminished pharmacological activity or even intoxications (Yamani et al., 2015; Cruz De Oliveira Costa et al., 2017; Ng et al., 2019) and may also cause adverse reactions that range from mild to life-threatening (Basak et al., 2019).

Among the different plant parts, the roots are most frequently used by informants for the treatment of worm infections in goats, followed by leaves. Yirga et al. (2012) reported that harvests involving roots, rhizomes, bulb, bark, stem or whole plant have a serious threat on the survival of the plant in its habitat; by contrast, the collection of leaves poses no significant threat to the survival of plants (Boadu and Asase, 2017). To foster sustainability, keepers could be encouraged to propagate the most useful trees or to concentrate on medicinally exploitable leaves, provided that only a reasonable amount of leaves is harvested (Canter et al., 2005). Such a management of resources would reduce eventual threats on interesting plant species. But the limited knowledge of tree species importance and the lack of appropriate propagation techniques remain major constraints in exploiting the domestication potential of medicinal plants (Amri and Kisangau, 2012). In the Haut-Katanga province, our data indicate that most of plants used in EV treatments are collected from the wild. This can be explained by the fact that keepers practice extensive farming in nature, far from cities and markets, and so have an easy access to collect plants in the wild.

All the cited remedies were monotherapies, i.e. based on preparations from a single plant; this may be related to previous experiences of keepers (Otieno et al., 2008) with plant species so efficient that they do not need additional constituents. But this finding markedly contrasts with previous ethnopharmacological study (Okombe et al., 2014a) in Kamina, a more remote Katanga region in which a series of multi-herbal recipes were reported. Maphosa and Masika (2010) reported that some herbalists are aware of the poisonous nature of certain recipes when it comes to mixing herbs; they are thus content with remedies prepared from a single plant. It is probable that the use of single or multi-plant recipes could be directly linked with the floristic diversity in the different study areas, a richer flora giving easier access to a diversity of plants and so to the possibility of testing, collecting and mixing species. In the province of Haut-Katanga, Meerts (2016) effectively observed markedly different phytogeographic and taxonomic assemblages in the three most important types of woody vegetation.

Similar to findings of Okombe et al. (2014a), the most common preparations of EV remedies include maceration and decoction in water for oral administration. Although keepers can use different routes of administration (Hilou et al., 2014; Okombe et al., 2014a, b), most of the treatments cited here were administered orally by drenching prepared remedy to the sick animal, by leaving the plants permanently in the drinker for some days or by mixing plant powder with food. The predominance of the oral route in the administration of treatments can be explained by the ease of administration and by the localization of the major symptoms observed; indeed, the oral route is by far the most common for administrating drugs intended to treat the gastrointestinal tract (Hua, 2020).

The dosage is far from standardized and is stated to depend on the experience of healers; this is generally the case in traditional medicine as exemplified previous ethnobotanical research in the study area (Okombe et al., 2014a, b).

The success of a treatment depends on many factors, including a correct diagnosis of the disease. This study indicates that goat keepers identify GIP infections from clinical signs (Table 2). Indeed, the definitive diagnosis of the disease is a major problem encountered by livestock keepers, leading them to rely solely on clinical signs (Cabaret et al., 2009; Fitzpatrick, 2013). These infections can lead to reduced primary digestion, decreased gastric secretions, dyspepsia, loss of plasma proteins, submandibular edema and blood loss (Craig, 2018; Vande Velde et al., 2018; Tufa et al., 2019); in severe cases, these symptoms can lead to death. Some of these clinical observations (presence of worms in stools, diarrhea, reduced growth, weight loss, anorexia, hypertrophy of mucosa) are indeed typical and recognizable signs. But it should be noted that many of the observed signs are common to several parasitic, bacterial or viral diseases. Although keepers stated that their experience guides the choice of administered “anthelmintic” treatment, the data we could collect were not precise enough to allow any distinction among cited plants.

Our results showed that the participants largely had their families as a source of traditional therapeutic knowledge. For this reason, Yirga et al. (2012) report that in some regions of the world, the transfer of knowledge about medicinal plants is done only from fathers to their first son or to another male child who could keep the secret; this could be the cause of the increase in the number of male livestock keepers. Elderly keepers and non-educated (Table 1) have more knowledge on medicinal plants and their uses, due to long direct contact with plant resources.

Literature data showed that some of the most cited plants, used by the goat keepers against GIP infection symptoms, have been reported to possess anthelmintic activities and some active chemical constituents have been identified. However, these properties have not yet been sufficiently explored for *V. fischeri* and *V. mombassae*. This is clearly an area that needs further investigation to validate the traditional use and to develop an eventual improved formulation that could be recommended as anthelmintic.

## 5 Conclusions and future directions

This study suggests that goat keepers in three territories of the Haut-Katanga province make use of ethnoveterinary knowledge and practices. The substitution or confusion observed in the identification of species is an aspect that warrants partcular attention as this may impact the expected pharmacological properties. It is also imperative to train farmers in correct propagation techniques to encourage the domestication of valuable and endangered medicinal lignous plants. This will create new opportunities for local people, providing alternative income and reducing pressure on the wild species population. Therefore, significant research into the chemical and biological properties of these lowly explored plants is absolutely needed to determine their anthelmintic efficacy and define their exact mechanism of action that could become important for the development of new anthelmintic drugs.

## Data Availability

The original contributions presented in the study are included in the article/Supplementary Material, further inquiries can be directed to the corresponding author.

## References

[B1] Ahmad M. Sultana S. Fazl-i-hadi S. Hadda T. Rashid S. Zafar M. (2014). An ethnobotanical study of medicinal plants in high mountainous region of Chail valley (District Swat-Pakistan). J. Ethnobiol. Ethnomed. 10, 36–18. 10.1186/1746-4269-10-36 24739524 PMC4022037

[B2] Alawa J. P. Jokthan G. E. Akut K. (2002). Ethnoveterinary medical practice for ruminants in the Sub-humid zone of Northern Nigeria. Prev. Vet. Med. 54, 79–90. 10.1016/s0167-5877(01)00273-2 12062521

[B3] Amjad M. S. Qaeem M. Ahmad I. Khan S. U. Chaudhari S. K. Malik N. Z. (2017). Descriptive study of plant resources in the context of the ethnomedicinal relevance of indigenous flora: A case study from toli peer national park, azad Jammu and Kashmir, Pakistan. PLoS One 12, e0171896–e0171897. 10.1371/journal.pone.0171896 28192466 PMC5305106

[B4] Amri E. Kisangau D. P. (2012). Ethnomedicinal study of plants used in villages around Kimboza forest reserve in Morogoro, Tanzania. J. Ethnobiol. Ethnomed. 8, 1–9. 10.1186/1746-4269-8-1 22221935 PMC3268735

[B5] Bakari A. Mwamba M. Lumbu S. Okusa P. Duez P. Kahumba B. (2017). Hypoglycemic and antihyperglycemic activities of nine medicinal herbs used as antidiabetic in the region of Lubumbashi (DR Congo). Phyther. Res. 31, 1029–1033. 10.1002/ptr.5814 28425214

[B6] Basak S. Aadi R. Parida A. Mitra S. Rangan L. (2019). Evaluation of rapid molecular diagnostics for differentiating medicinal *Kaempferia* species from its adulterants. Plant divers. 41, 206–211. 10.1016/j.pld.2019.04.003 31453420 PMC6704042

[B7] Boadu A. A. Asase A. (2017). Documentation of herbal medicines used for the treatment and management of human diseases by some communities in southern Ghana. Altern. Med. 2017, 1–12. 10.1155/2017/3043061 PMC5480049 28684965

[B8] Cabaret J. Benoit M. Laignel G. Nicourt C. (2009). Current management of farms and internal parasites by conventional and organic meat sheep French farmers and acceptance of targeted selective treatments. Vet. Parasitol. 164, 21–29. 10.1016/j.vetpar.2009.04.018 19414221

[B9] Canter P. H. Thomas H. Ernst E. (2005). Bringing medicinal plants into cultivation: Opportunities and challenges for biotechnology. Trends Biotechnol. 23, 180–185. 10.1016/j.tibtech.2005.02.002 15780709

[B10] Chiribagula B. V. Amuri B. S. Kalonji M. S. Byanga K. J. Duez P. Simbi L. J. B. (2017). Étude ethnobotanique, phytochimique et évaluation de l’activité antiplasmodiale de 13 plantes réputées antipaludéennes dans la commune de Kenya (Lubumbashi, RDC). Phytotherapie, 1–10. 10.1007/s10298-017-1152-x

[B11] CJB (2012). African Plant Database – conservatoire et Jardin botaniques de la ville de Genève (CJB). Sandton: South African Natl. Biodivers. Inst.

[B12] Craig T. M. (2018). Gastrointestinal nematodes, diagnosis and control. Vet. Clin. NA Food Anim. Pract. 34, 185–199. 10.1016/j.cvfa.2017.10.008 29421029

[B13] Cruz De Oliveira Costa V. Borghi A. A. Mayer J. L. S. Sawaya A. C. H. F. (2017). Comparison of the morphology, anatomy, and chemical profile of *Mikania glomerata* and *Mikania laevigata* . Planta Med. 84, 191–200. 10.1055/s-0043-119226 28926862

[B14] Dansou C. C. Olounladé P. A. Konmy B. S. B. Songbé O. Arigbo K. B. Aboh A. B. (2021). Ethno-veterinary survey and quantitative study of medicinal plants with anthelmintic potential used by sheep and goat breeders in the Cotton zone of Central Benin (West Africa). Multidiscip. Sci. J. 4, 544–563. 10.3390/j4040040

[B15] De Weerdt J. Toirambe B. Verhegghen A. Defourny P. Beeckman H. (2018). “La végétation de la province du Haut-Katanga,” in Haut-Katanga: Lorsque richesses économiques et pouvoirs politiques forcent une identité régionale- Tome 1_Cadre naturel, peuplement et politique. Editors Ngoy P. K. Badie M. K. Krawczyk J. Laghmouch M. Tshonda J. O. (Belgium: Musée royal de l’Afrique centrale), 31–54.

[B16] Fitzpatrick J. L. (2013). Global food security – The impact of veterinary parasites and parasitologists. Vet. Parasitol. 2, 233–248. 10.1016/j.vetpar.2013.04.005 23622818

[B17] Gagman A. H. Ahmad H. Ahmad N. Izzaudin I. Him N., 2019. The efficacy of *Vitex doniana* and *Boswellia dalzielii* against egg hatch of *Caenorhabditis elegans* . Bayero J. Pure Appl. Sci. 12, 737–742. doi: doi: 10.4314/bajopas.v12i1.110S

[B19] Gemeda B. A. Amenu K. Magnusson U. Dohoo I. Hallenberg G. S. Alemayehu G. (2020). Antimicrobial use in extensive smallholder livestock farming systems in Ethiopia: Knowledge, attitudes, and practices of livestock keepers. Front. Vet. Sci. 7, 55–15. 10.3389/fvets.2020.00055 32175334 PMC7055293

[B21] Herrero M. Grace D. Njuki J. Johnson N. Enahoro D. Silvestri S. (2013). The roles of livestock in developing countries. Animal 7, 3–18. 10.1017/S1751731112001954 23121696

[B22] Hilou A. Rappez F. Duez P. (2014). Ethnoveterinary management of cattle helminthiasis among the fulani and the mossi (Central Burkina Faso): Plants used and modes of use. Int. J. Biol. Chem. Sci. 8, 2207–2221. 10.4314/ijbcs.v8i5.24

[B23] Hua S. (2020). Advances in oral drug delivery for regional targeting in the gastrointestinal tract - influence of physiological, pathophysiological and pharmaceutical factors. Front. Pharmacol. 11, 524. 10.3389/fphar.2020.00524 32425781 PMC7212533

[B25] Kalenga H. K. Vandeput S. Antoine-Moussiaux N. Moula N. Kashala J.-C. K. Farnir F. (2015). Goat breeding in Lubumbashi (DRC): 1. Principal component analysis of linear measurements of local population. Livest. Res. Rural. Dev. 27, 12. Available at: https://www.researchgate.net/publication/286875990 .

[B26] Klapwijk C. J. Schut M. van Asten P. J. A. Vanlauwe B. Giller K. E. Descheemaeker K. (2020). Micro-livestock in smallholder farming systems: The role, challenges and opportunities for cavies in south kivu, eastern DR Congo. Trop. Anim. Health Prod. 52, 1167–1177. 10.1007/s11250-019-02112-9 31758384 PMC7190603

[B27] Lengbiye E. M. Ngbolua K.-N. Messi L. M. Rasoazanany E. O. Luyeye F. L. Mbing J. N. (2020). Microscopic studies, mineral composition and bioactivity of *Vitex madiensis* microscopic studies, mineral composition and bioactivity of *Vitex madiensis* Oliv. (Lamiaceae). Discov. Phytomedicine 7, 177–185. 10.15562/phytomedicine.2020.149

[B28] Lengbiye E. M. Ngbolua K. Bongo G. N. Messi M. Noté O. P. Mbing J. N. (2018). *Vitex madiensis* Oliv. (Lamiaceae): Phytochemistry, pharmacology and future directions, a mini-review. J. Pharmacogn. Phytochem. 7, 244–251.

[B29] Malaisse F. (1997). Se nourrir en forêt africaine, Les presse. Gembloux: CTA, 384.

[B30] Maphosa V. Masika P. J. (2010). Ethnoveterinary uses of medicinal plants: A survey of plants used in the ethnoveterinary control of gastro-intestinal parasites of goats in the eastern cape province, south Africa. Pharm. Biol. 48, 697–702. 10.3109/13880200903260879 20645744

[B31] Meerts P. (2016). An annotated checklist to the trees and shrubs of the upper Katanga (D.R. Congo). Phytotaxa 258, 201–250. 10.11646/phytotaxa.258.3.1

[B32] Mpofu T. J. Nephawe K. A. Mtileni B. (2020). Prevalence of gastrointestinal parasites in communal goats from different agro-ecological zones of South Africa. Vet. World 13 (1), 26–32. 10.14202/vetworld.2020.26-32 32158147 PMC7020112

[B33] Mukazayire M. Minani V. Ruffo C. K. Bizuru E. Stévigny C. Duez P. (2011). Traditional phytotherapy remedies used in Southern Rwanda for the treatment of liver diseases. J. Ethnopharmacol. 138, 415–431. 10.1016/j.jep.2011.09.025 21963560

[B34] N’Danikou S. Achigan-dako E. G. Tchokponhoue D. A. Agossou C. O. A. Houdegbe C. A. Vodouhe R. S. (2015). Modelling socioeconomic determinants for cultivation and *in-situ* conservation of *Vitex doniana* Sweet (Black plum), a wild harvested economic plant in Benin. J. Ethnobiol. Ethnomed. 11, 28–16. 10.1186/s13002-015-0017-3 25925635 PMC4422408

[B35] Ng W. Y. Hung L. Y. Lam Y. H. Chan S. S. Pang K. S. Chong Y. K. (2019). Poisoning by toxic plants in Hong Kong: A 15-year review. Hong Kong Med. J. 25, 102–112. 10.12809/hkmj187745 30967518

[B36] Ngona I. A. (2008). “Performances et facteurs d’influence de la reproduction de l’espèce caprine en milieu tropical,” in Thèse d’agrégation en médecine vétérinaire (DR Congo: Université de Lubumbashi), 165.

[B37] Novotna B. Polesny Z. Pinto-Basto M. F. Van Damme P. Pudil P. Mazancova J. (2010). Proximate composition, phenolic content and antioxidant activities of three black plum (*Vitex* sp.) fruits: Preliminary results. J. Food Technol. 8, 118–125. 10.3923/jftech.2010.118.125

[B38] Okombe E. V. (2011). Activité antihelminthique de la poudre d'écorce de racine de *Vitex thomasii* De Wild (Verbenaceae) sur *Haemonchus contortus* chez la chèvre. These d’agrégation en medecine vétérinaire. DR Congo: Université de Lubumbashi, 242.

[B39] Okombe E. V. Lumbu Simbi J.-B. Stévigny C. Vandenput S. Pongombo Shongo C. Duez P. (2014a). Traditional plant-based remedies to control gastrointestinal disorders in livestock in the regions of Kamina and Kaniama (Katanga province, Democratic Republic of Congo). J. Ethnopharmacol. 153, 686–693. 10.1016/j.jep.2014.03.027 24657601

[B40] Okombe E. V. Pongombo C. S. Duez P. Vandenput S. (2014b). Remèdes vétérinaires traditionnels utilisés dans les élevagesdechèvres à Lubumbashi et proche périphérie, RD Congo. Phytothérapie 12, 234–241. 10.1007/s10298-014-0873-3

[B41] Otieno J. N. Hosea K. M. M. Lyaruu H. V. Mahunnah R. L. A. (2008). Multi-plant or single-plant extracts, which is the most effective for local healing in Tanzania? Afr. J. Tradit. Complement. Altern. Med. 5, 165–172. 10.4314/ajtcam.v5i2.31269 20161933 PMC2816543

[B42] Stuessy T. F. Sohmer S. H. (1996). Sampling the green world: Innovative concepts of collection, preservation, and storage of plant diversity. New York: FAO-AGRIS - Columbia U, 289.

[B43] Suleiman M. M. Yusuf S. (2008). Antidiarrheal activity of the fruits of *Vitex doniana* in laboratory animals. Pharm. Biol. 46, 387–392. 10.1080/13880200802055826

[B44] Tufa S. S. Sölkner J. Mészáros G. Haile A. Mwacharo J. Khayatzadeh N. (2019). Indigenous knowledge, practices and preferences in control of gastrointestinal nematodes in Bonga and Horro sheep of Ethiopia. Small Rumin. Res. 175, 110–116. 10.1016/j.smallrumres.2019.04.019

[B46] Vande Velde F. Charlier J. Claerebout E. (2018). Farmer behavior and gastrointestinal nematodes in ruminant livestock — uptake of sustainable control approaches. Front. Vet. Sci. 5, 255. 10.3389/fvets.2018.00255 30386785 PMC6198092

[B47] Wasso D. S. Akilimali J. I. Baenyi P. Bajope J. B. (2018). Goat farming: Current situation, challenges and socio-economic impact on the population of the territory of walungu, democratic republic of Congo. J. Appl. Biosci. 129 (1), 13050–13060. 10.4314/jab.v129i1.8

[B48] Waterman C. Smith R. A. Pontiggia L. Dermarderosian A. (2010). Anthelmintic screening of Sub-Saharan African plants used in traditional medicine. J. Ethnopharmacol. 127, 755–759. 10.1016/j.jep.2009.11.025 19962435

[B49] WMA (2015). World Medical Association Declaration of Helsinki: Ethical principles for medical research involving human subjects. JAMA 310, 2015. 10.1001/jama.2013.281053 24141714

[B50] Yamani A. Bunel V. Antoine M. Husson C. Stévigny C. Duez P. (2015). Substitution between *aristolochia* and *bryonia* genus in north-eastern Morocco: Toxicological implications. J. Ethnopharmacol. 166, 250–260. 10.1016/j.jep.2015.03.036 25797117

[B51] Yirga G. Teferi M. Gidey G. Zerabruk S. (2012). An ethnoveterinary survey of medicinal plants used to treat livestock diseases in Seharti-Samre district, Northern Ethiopia. Afr. J. Plant Sci. 6, 113–119. 10.5897/AJPS11.242

[B52] Zvinorova P. I. Halimani T. E. Muchadeyi F. C. Matika O. Riggio V. Dzama K. (2016). Prevalence and risk factors of gastrointestinal parasitic infections in goats in low-input low-output farming systems in Zimbabwe. Small Rumin. Res. 143, 75–83. 10.1016/j.smallrumres.2016.09.005 27766016 PMC5063533

