# Supplementary material for: Smallholders’ knowledge about healing goat gastrointestinal parasite infections with wild plants in southern DR Congo
Source: Front Pharmacol. 2023 Mar 1;14:1124267. doi: 10.3389/fphar.2023.1124267 (PMC10016610; doi:10.3389/fphar.2023.1124267)
Supplement: Supplementary file 1 [file Presentation1.pdf]

Supplementary Material

Smallholders' knowledge about healing goat gastrointestinal parasite infections with wild plants in Southern DR Congo

Gaël Nzuzi Mavungu*, Cedrick Shakalenga Mutombo, Désiré Mujike Numbi, Salvatora Nkulu Nsenga, Welcome Nonga Muyumba, Celestin Shongo Pongombo, Salvius Amuri Bakari, Amandine Nachtergael, Sandrina Vandenput, Victor Embeya Okombe, Pierre Duez

*** Correspondence:** mavungug@unilu.ac.cd

Questionnaire survey for data collection on ethnoveterinary practices from goat farmers

1. Place (District, Village) :
2. Tribal group/caste :
3. Identity of the respondent :

- Number (following the order of the respondents) :
- Sex :
- Age :
- Educational status : University/ Secondary education/ illiterate/ elementary school.
- Source of ethnoveterinary healing knowledge :
- Ethnoveterinary practice experience (year) :

4. What do you know about gastrointestinal parasite infection and how do you diagnose them in your sick animals? (symptoms?)
5. How do you manage these disorders?

- Name and type of the plant :
- Parts/derivatives used as medicine :
- Is there any combination with other species/proportion :
- Preparation method(s) :
- Administration form (s) :
- Approximate dosage and other information :

6. Any other information not covered above?

Reporter name :

Date :

Plant pictures

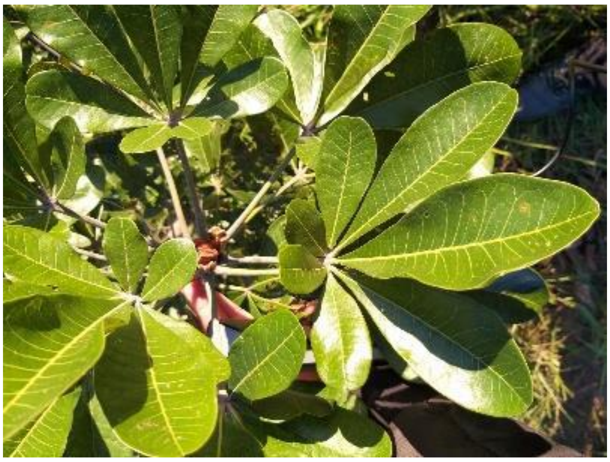

1: *Oldfieldia dactylophylla* (Welw. ex Oliv.) J.Leonard

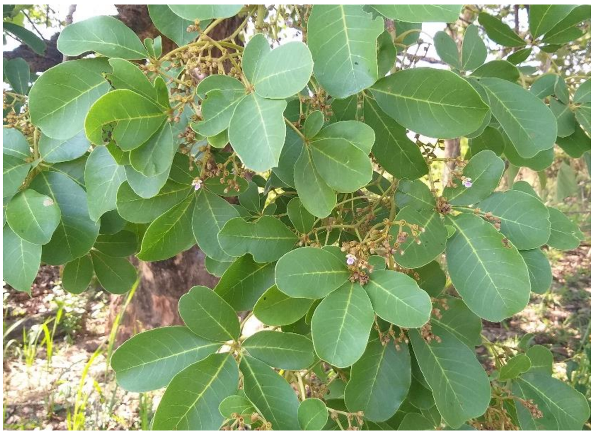

2: *Vitex doniana* Sweet

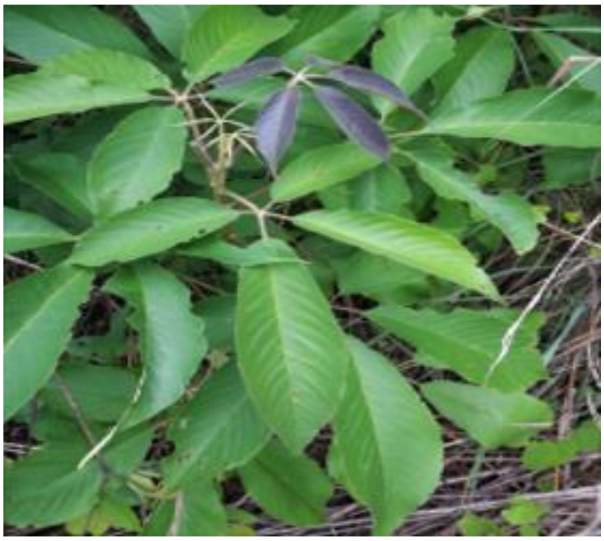

3 : *Vitex fischeri* Gürke

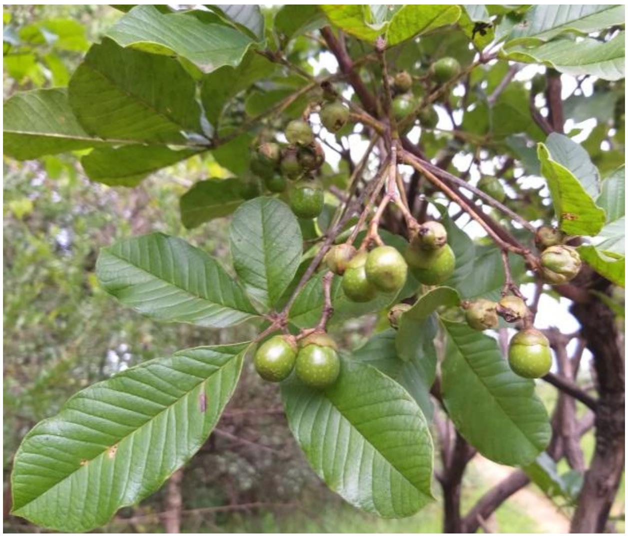

4 : *Vitex madiensis* Oliv. subsp. *milanjiensis* (Britten) F.White

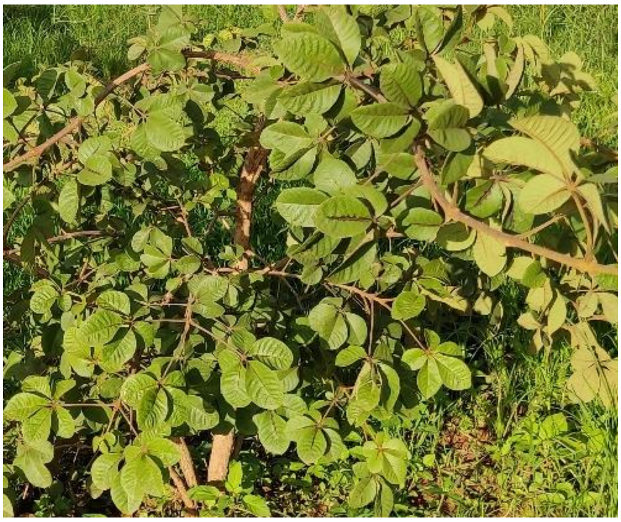

5 : *Vitex mombassae* Vatke
